# Identification of volatile compounds from bacteria by spectrometric methods in medicine diagnostic and other areas: current state and perspectives

**DOI:** 10.1007/s00253-021-11469-7

**Published:** 2021-08-20

**Authors:** Nils Kunze-Szikszay, Maximilian Euler, Thorsten Perl

**Affiliations:** 1 Department of Anesthesiology, University Medical Center Göttingen, Robert-Koch-Straße 40, 37075 Göttingen, Germany; 2 Department of General, Visceral and Pediatric Surgery, University Medical Center Göttingen, Robert-Koch-Straße 40, 37075 Göttingen, Germany

**Keywords:** Bacteria, Volatile organic compounds, Spectrometry, Breath analyses, Diagnostics, Mass spectrometry, Ion mobility spectrometry

## Abstract

**Abstract:**

Diagnosis of bacterial infections until today mostly relies on conventional microbiological methods. The resulting long turnaround times can lead to delayed initiation of adequate antibiotic therapy and prolonged periods of empiric antibiotic therapy (e.g., in intensive care medicine). Therewith, they contribute to the mortality of bacterial infections and the induction of multidrug resistances. The detection of species specific volatile organic compounds (VOCs) emitted by bacteria has been proposed as a possible diagnostic approach with the potential to serve as an innovative point-of-care diagnostic tool with very short turnaround times. A range of spectrometric methods are available which allow the detection and quantification of bacterial VOCs down to a range of part per trillion. This narrative review introduces the application of spectrometric analytical methods for the purpose of detecting VOCs of bacterial origin and their clinical use for diagnosing different infectious conditions over the last decade.

**Key Points:**

*• Detection of VOCs enables bacterial differentiation in various medical conditions.*

*• Spectrometric methods may function as point-of-care diagnostics in near future.*

## Introduction

In 1921, 100 years before this manuscript was put together, Harper F. Zoller and W. Mansfield Clark published a bench study on the production of volatile fatty acids by dysenteric bacteria and the influence of different growing conditions on their production (Zoller and Clark 1921). By the use of distillation, they directly verified the types and amounts of fatty acids produced by several bacterial strains and concluded that those might significantly contribute to the “unpleasant symptoms” which correlated with bacterial infections of the human intestinal tract. Most likely, neither of them was thinking about the possibility of detecting volatile organic compounds (VOCs) as an option for the diagnosis of infectious diseases. Nowadays, with analytical methods allowing the detection of lowest quantities of VOCs in a headspace or exhaled breath sample, non-invasive diagnostic tools based on gas analytics seem to be within reach.

**Table 1:** List of studies discussed in this review that include clinical application of analytical methods

**Study**	**Year**	**Sample size**	**Setting**	**Conditions and pathogens studied**	**Analytical method**	**Sample matrix**	**Sample method**
**Respiratory infections**							
Nasir et al	2018	60	Single-center, UK	Cystic fibrosis (*P. aeruginosa*, *S. aureus*)	GCxGC-TOF–MS	BAL^1^	Direct analysis
Coronel Teixeira et al	2017	106	Single-center, Paraguay	Tuberculosis (*M. tuberculosis*)	e-nose	Breath	Direct analysis
Zetola et al	2017	71	Single-center, Botswana	Tuberculosis (*M. tuberculosis*)	e-nose	Breath	Pre-collected, sampling bags
Van Oort et al	2017	93	Single-center, Netherlands	VAP^2^	GC–MS	Breath	Pre-collected, sampling bags
Gao et al	2016	60	Single-center, PR China	VAP^2^ (*A. baumanii*)	GC–MS	Breath	Pre-collected, desorption tubes
Neerincx et al	2016	18	Single-center, Netherlands	Cystic fibrosis (*S. aureus*)	GC–MS	Breath	Pre-collected, sampling bags
Sahota et al	2016	21	Single-center, UK	Tuberculosis (*M. tuberculosis*)	FAIMS	Breath	Pre-collected, sampling bags
Fowler et al	2015	46	Single-center, UK	Lower airway infections	GC–MS	Breath	Pre-collected, desorption tubes
Kramer et al	2015	11	Single-center, Germany	Cystic fibrosis (*P. aeruginosa*, *S. aureus*, *C. albicans*, *A. xylososidans*)	GC–MS	Breath	SPME
Schnabel et al	2015	100	Single-center, Netherlands	VAP^2^	GC-TOF–MS	Breath	Pre-collected, sampling bags
Filipiak et al	2014	28	Single-center, Austria	VAP^2^ (*S. aureus*, *C. albicans*, *E. coli*)	GC-TOF–MS	Breath	Pre-collected, desorption tubes
Nakhleh et al	2014	198	Multi-center, Rep. of South Africa	Tuberculosis (*M. tuberculosis*)	e-nose	Breath	Pre-collected, desorption tubes
Gilchrist et al	2013	20	Single-center, UK	Cystic fibrosis (*P. aeruginosa*, *S. aureus*)	SIFT-MS	Breath	Direct analysis
Goeminne et al	2012	28	Single-center, Belgium	Cystic fibrosis (*P. aeruginosa*)	GC–MS	Sputum	SPME
**Gastrointestinal infections**							
Berkhout et al	2019	843	Multi-center, Netherlands/Belgium	Neonatal late-onset sepsis	FAIMS	Stool	Direct headspace sampling
Patel et al	2019	106	Single-center, UK	CDI^4^ (*C. difficile*)	GC-TOF–MS	Stool	Pre-collected headspace, desorption tubes
Arasaradnam et al	2016	76	Single-center, UK	IBD^3^	FAIMS	Breath	Pre-collected, sampling bags
Bromers et al	2015	213	Two-center, Netherlands	CDI^4^ (*C. difficile*)	FAIMS	Stool	Direct headspace sampling
Arasaradnam et al	2013	62	Single-center, UK	IBD^3^	e-nose, FAIMS	Urine	Direct headspace sampling
Garner et al	2009	9	Single-center, Bangladesh	Cholera (*V. cholera*)	GC–MS	Stool	SPME
Lechner et al	2005	25	Single-center, Austria	Gastritis (*H. pylori*)	PTR-MS	Breath	Pre-Collected, Sampling bags
**Bloodstream infections**							
Zhong et al	2019	46	Single-center, PR China	BSI^5^ (*E. coli*)	CDI-MS	Blood	SPME
Chingin et al	2015	130	Single-center, PR China	BSI^5^ (*S. aureus*, *E. coli*, *K. pneumoniae*, *A. baumanii*, *P. aeruginosa*)	APCI-MS	Blood	Direct headspace sampling
Other infections							
Daulton et al	2020	19	Single-center, UK	Wound infection	GC-IMS	Wound dressing	Direct headspace sampling
Lacey et al	2020	243	Single-center, UK	Maternal streptococcal colonization (*group B streptococci*)	GC-IMS	Vaginal swabs	Direct headspace sampling
Kviatkovski et al	2018	26	Single-center, Israel	Otitis externa (*P. aeruginosa*)	GC–MS	Pus samples	Pre-collected, desorption tubes
Blankenstein et al	2015	57	Single-center, Germany	Bacterial vaginosis	IMS	Vaginal swabs	Direct headspace sampling
Roine et al	2014	101	Single-center, Finland	UTI^6^ (*E. coli*, *S. saprophyticus*, *E. faecalis*, *Klebsiella* spp.)	e-nose	Urine	Direct headspace sampling
Chaim et al	2003	174	Single-center, Israel	Bacterial vaginosis	IMS	Vaginal swabs	Direct headspace sampling

^1^*BAL* bronchoalveolar lavage; ^2^*VAP* ventilator-associated pneumonia; ^3^*IBD* inflammatory bowel disease; ^4^*CDI Clostridoides difficile* infection; ^5^*BSI* bloodstream infection; ^6^*UTI* urinary tract infection

Bacterial infections and the rise of multidrug resistances significantly contribute to morbidity and mortality worldwide. Mortality rates in septic shock, for which bacterial infections are the leading cause, do rise above 50%, even if patients are treated in a modern intensive care unit (SepNet Critical Care Trials Group 2016). Early diagnosis and pathogen identification are crucial for the treatment of bacterial infections. The early beginning and the adequacy of the initial antibiotic therapy significantly contribute to the patient’s outcome (Kumar et al. 2009). However, no biomarkers are available that reliably predict the onset of bacterial infections or sepsis. Diagnosis therefore remains clinical and needs to rely on potentially life-threatening symptoms of the patients (Singer et al. 2016). Until today, culturing is the standard method for the identification of causative pathogens in medical microbiology. The use of these highly sensitive and very specific, but also time-consuming techniques, leads to turnaround times of up to 3 days for final results (Tabak et al. 2018). During this time, empiric broad-spectrum antibiotic therapy is used to treat the infection (Paul et al. 2010). Without identification of the infecting organism and its susceptibility, this empiric (or calculated) antibiotic therapy potentially risks inadequate treatment and induction of drug resistances and adverse effects (MacFadden et al. 2014).

The availability of rapid methods for the diagnosis of bacterial infections and identification of the causative pathogen would help narrowing the empiric window and therewith contribute to a safer and more effective antibiotic therapy regimen (Battle et al. 2017; Tumbarello et al. 2010). Innovative applications include microbial identification directly from positive blood cultures by the use of matrix-assisted laser desorption ionization-time-of-flight mass spectrometry (MALDI-TOF MS) or the Accelerate Pheno™ System, which combines fluorescence in situ hybridization (FISH) with morphokinetic cellular analysis (MCA) (Pancholi et al. 2018; Rodriguez-Sanchez et al. 2014). These proteomic (MALDI-TOF MS) and optical (FISH-MCA) methods contribute to shorter turnaround times and were shown to deliver reliable results on pathogen identification and antibiotic resistances. Both systems are commercially available and in clinical use. However, both methods require at least a positive blood culture (or equivalent specimen) as a starting point, which remains a limiting factor regarding turnaround times. They need to be operated by trained personnel in specialized microbiological laboratories which, together with the high costs for acquisition and operation, leads to centralization of these systems at large hospitals or commercial providers (Ratiu et al. 2017). Hospitals lacking such facilities may send their microbiological specimen to such laboratories, again increasing turnaround times is increased and in some instances risking the integrity of the specimen, depending on length and circumstances of the transport.

Multiplex polymerase chain reaction (multiplex PCR) is another innovative method allowing rapid pathogen identification including the detection of genetic antibiotic resistance markers. Currently, three systems (SepsiTest [Molzym Molecular Diagnostics, Bremen, Germany], IRIDICA BAC BSI [Abbott Diagnostics, Lake Forest, USA], LightCycler SeptiFast [Roche, Risch-Rotkreuz, Switzerland]) are commercially available for pathogen identification from blood cultures (Stevenson et al. 2016). One cartridge system (Unyvero A50 [Curetis, Holzgerlingen, Germany]) is targeting several localized infection sides as well as bloodstream infections (Burrack-Lange et al. 2018). Of those, the Unyvero A50 is the only system that can be operated point-of-care, which leads to relevant reduction of turnaround times (Kunze et al. 2015). However, multiplex PCR devices are not part of the clinical routine in the majority of hospitals and possess some methodological challenges by themselves. Depending on the composition of the pathogen and resistance marker panel of the devices, it may, for instance, remain unclear for the user whether a detected resistance marker is associated with the detected pathogen or with a resident organism that is not part of the pathogen panel. The mecA gene is a marker for methicillin resistance and occurs in both *Staphylococcus aureus* and *Staphylococcus epidermidis* and is an example for such a possible cause of misinterpretation (Becker et al. 2006). Multiplex PCR systems incur relevant costs, and data on their ability to reduce mortality, morbidity, and therapy costs remain inconsistent (Warhurst et al. 2015). Their use will therefore be limited to highly equipped hospitals in economically developed countries.

The detection of VOCs of microbial origin is an alternative approach with the potential to be a robust, fast, and relatively low-cost point-of-care method for pathogen differentiation and identification. A variety of analytical methods allows the detection and identification of VOCs (Ratiu et al. 2017). There is growing knowledge on the occurrence and composition of volatile metabolites emitted from bacteria and other microbes (Bos et al. 2013; Schulz and Dickschat 2007). With this narrative review, we aim to give a brief overview on the current state of knowledge on the use of spectrometric methods for the diagnosis of bacterial infections and possible perspective of their future use.

## Spectrometric methods for VOC detection

The metabolic pathways of bacteria have been investigated in detail (Schulz and Dickschat 2007). Ideally, the occurrence of a VOC should be assignable to a known metabolic pathway. However, the production of VOCs by bacteria is influenced by their growing conditions (O'Hara and Mayhew 2009). Our knowledge of bacterial metabolic pathways and therewith VOC production under various conditions in the human body is still very limited. In 2013, Bos et al. published a detailed review of VOCs produced by the six most relevant bacteria in sepsis: *S. aureus*, *S. pneumoniae*, *E. faecalis*, *K. pneumoniae*, *P. aeruginosa*, and *E. coli* (Bos et al. 2013).

The analytical challenge for available devices is the detection and quantification of VOCs in a complex matrix of gaseous samples with traces down to parts per billion (ppb) or even trillion (ppt). Ideally, the detected VOCs are produced by species cultivated on defined, standardized substrate such as culture media. In the clinical context, it may also be desirable to directly detect VOCs from highly diverse substrates like urine, feces, sputum, vaginal fluids, tissues, or breath (Amann et al. 2014; Eng et al. 2015; Lacey et al. 2020; Purkhart et al. 2011; Ratiu et al. 2019).

Gas chromatography coupled with mass spectrometry (GC–MS) is the gold standard for VOC detection (Ratiu et al. 2017). GC–MS does possess large databases for identification of substances and the capability of separating and unequivocal identification of compounds. Multiple publications in the field of bacterial VOC determination with GC–MS demonstrated its applicability for this purpose (Liebeke et al. 2012; Tait et al. 2014). Alternatives to GC–MS are other mass spectrometric instrumentations like proton transfer reaction-mass spectrometers (PTR-MS) or selected ion flow tube-mass spectrometer (SIFT-MS). Secondary electrospray ionization mass spectrometry (SESI-MS) has been shown to be more sensitive compared to PTR-MS or SIFT-MS enabling substance detection in the ppt range (Woolfenden 2010). A strength of these devices, which are still bulky and not portable, is the possibility of real-time measurements at a high sensitivity in range of ppb. However, a chemical identification of unknown VOCs is not possible.

Immobility due to bulky setup and need for high effective vacuum pumps and purified gas supply is a common drawback of all mass spectrometric devices. Thus, gas samples for diagnostic purposes need be transferred to the device for offline analysis. Gas bags or absorptive mediums like needle traps and solid-phase microextraction tools are used to transfer the sample to the mass spectrometric device. These pre-concentration techniques possibly influence compound composition of gas samples by selective absorption and desorption depending on characteristics of the chosen transfer material (Slingers et al. 2021). Therefore, a favorable approach to determine VOCs is not only to measure real-time but also in a point-of-care setting with the possibility of direct sampling at the patient’s bed side (e.g., breath sampling).

In contrast to mass spectrometric methods, ion mobility spectrometry (IMS) applications are portable and even available as handhelds. IMS devices are already widely used by rescue services, security authorities, and the military for the detection of hazardous substances, explosives, and chemical warfare agents (Cumeras et al. 2015). The method allows substance detection and quantification down to a range of ppt (Kunze et al. 2015). Time-of-flight-IMS (TOF-IMS), aspiration-IMS (aIMS), field asymmetric ion mobility spectrometry (FAIMS), and differential mobility spectrometers IMS (DMS) have been described for microbiological VOC detection (Costanzo et al. 2017; Ratiu et al. 2017). IMS devices can be used separately (e.g., as screening tools for a known substance), or they can be coupled with a chromatographic method, which allows analyses of more complex gas samples. GC columns or multicapillary columns (MCC) are mostly used for pre-separating complex matrices such as breath or headspace samples of complex growth media (Baumbach 2009; Speckbacher et al. 2021). IMS devices are inexpensive, robust, and portable. They therefore have the potential to serve as point-of-care diagnostic tools in challenging healthcare settings.

The spectrum of IMS-based analytical methods is growing. Couplings of more than one detector (e.g., FAIMS-FAIMS), the use of different pre-separation methods (e.g., GC, MCC), different ionization methods, and even pre-concentration techniques facilitate individual analytical solutions for specific circumstances. However, due to diverse setup of these systems, a comparison of findings is difficult. Targeted analyses are possible when known substances are investigated, and reference measurements can be provided for confirmation. The composition of volatile organic compounds over growing bacterial species, however, contains mostly substances that are unknown to the researcher in the first place. This untargeted analytic approach often leads to the approach of pattern recognition, where none or not all substances are identified. Using IMS techniques, the identification of (suspected) substances can be very challenging and time- and resource-consuming. The position of a substance in the topogram of a GC-IMS device is unique, but it needs to be confirmed by reference measurements of the pure substance. Once a substance is confirmed, this information can contribute to a specific database for each application, allowing identification of substances even without a reference measurement. However, this information is only valid for very similar IMS applications. The diversity of analytical setups of devices makes it often hard to compare results and to deduce conclusions from results of similar but “different enough” methods.

Electronic noses, often referred to as “e-noses”, represent a third analytical approach by using biochemical sensor arrays for the detection of VOCs and VOC patterns (Wilson 2020). E-noses are small and inexpensive and allow screening for certain chemical conditions while being easy to operate. However, these devices do not allow identification of individual substances. It is therefore necessary to configure a suitable sensor array for each individual analytical question. E-nose applications may be used to translate the results of more sophisticated analytical methods, like mass spectrometric or IMS methods, into easy-to-operate and rugged bedside tools.

## Diagnostic applications for bacterial infections

Over the last two decades, there is a growing interest in the experimental and clinical application of spectrometric methods for the detection of bacterial VOCs for diagnostic purposes. The results of the studies from a broad variety of medical fields underline the potential of the approach. Following, we aim to give a comprehensive overview of different applications with a focus on clinical studies published within the last 5 years (Table 1).

### Respiratory infections

Tuberculosis (TB), caused by *Mycobacterium tuberculosis*, is one of the leading causes of death among infectious diseases worldwide (Khatua et al. 2017). A reliable, simple, and cheap test for the diagnosis of TB is of outmost interest, especially for low-income countries. In 2016, Sahota et al. described the use of a FAIMS device with a non-radioactive UV ionization source for VOC pattern recognition to diagnose TB in the UK (Sahota et al. 2016). They investigated a rather small number of 21 breath samples of TB patients compared to 19 healthy controls and found a sensitivity and specificity of 81% and 79%. Three groups used e-nose devices for the same purpose and described sensitivities and specificities of 88–94% and 90–93% for diagnosing TB (Coronel Teixeira et al. 2017; Nakhleh et al. 2014; Zetola et al. 2017). None of the four studies identified individual VOCs that were used for the prediction of TB.

The majority of pulmonary infections in cystic fibrosis (CF) is caused by *Pseudomonas aeruginosa* (PA) or *Staphylococcus aureus* (SA). In 2016, Neerincx et al. published a feasibility study on the identification of SA-infected CF patients in a collective of 18 patients. Using Tedlar® bags, they collected breath samples and analyzed them using GC–MS. They were able to identify nine VOCs which were useful for separating SA-infected patients. Three of these VOCs did significantly differ between the groups (1,4 pentadiene, acetone, undecane). Interestingly, seven of the 13 CF patients infected with SA were also infected with PA, *Serratia marcescens*, *or Haemophilus influenzae* (Neerincx et al. 2016). By measuring the concentration of hydrogen cyanide using SIFT-MS analyses of nose-exhaled breath, Gilchrist et al. successfully differentiated between chronically PA-infected and non-infected CF patients (Gilchrist et al. 2013). The discrimination of PA-infected and non-infected CF patients is also possible by pattern recognition of VOCs as it was shown in a study using SPME enrichment for indirect sampling of breath for GC–MS analyses from CF patients (Kramer et al. 2015). Besides breath analysis, detection of PA-specific VOCs was also detected in sputum headspace (Goeminne et al. 2012) and over bronchoalveolar lavage fluid samples (Nasir et al. 2018).

Ventilator-associated pneumonia (VAP) is a common nosocomial infection. Diagnosis is time critical and still based on unspecific clinical criteria and pathogen detection in bronchoalveolar lavage. Breath analysis could be a feasible, non-invasive approach for both VAP diagnosis and pathogen identification. Schnabel et al. in 2015 used GC-TOF–MS for investigating samples of exhaled breath from 100 ventilated patients in intensive care for the occurrence of VAP-specific VOCs. With a set of 12 VOCs, they were able to correctly discriminate between patients with and without VAP (Schnabel et al. 2015). Other authors described changes in the volatile metabolite profile of ventilated patients with bacterial colonization of the lower respiratory tract (Fowler et al. 2015). In 2017, van Oort et al. did also discriminate VAP from control with relatively good accuracy. They analyzed 12 VOCs that changed during pneumonia and 52 VOCs that changed in colonized patients (van Oort et al. 2017). Filipiak et al. showed that the concentration of specific compounds changed over the course of the infection and also correlated with illness severity (Filipiak et al. 2015). In 2016, Gao et al. published a study in which they differentiated the presence from the absence of *A. baumanii* in the lower respiratory tract, as well as the colonization from an actual infection using breath VOC profiles (Gao et al. 2016).

### Gastrointestinal infections

Another potential application is the investigation of fecal volatilome for the diagnosis of gastrointestinal infections. One confounding factor for such analyses is the highly inter-individual fecal microbiome. Together with a complex volatile background caused by fermentation processes, this leads to a challenging environment for gas analyses (Elmassry and Piechulla 2020).

*Clostridioides difficile* causes antibiotic associated diarrhea and has been found to cause a unique fecal volatilome. Both GC-tof–MS and FAIMS discriminated between (unprocessed) infected and uninfected stool samples with high diagnostic accuracy (Bomers et al. 2015; Patel et al. 2019). GC–MS has also been successfully used to identify volatile biomarkers of *Vibrio cholorae* emitted from stool samples (Garner et al. 2009).

A prospective multicenter study from the Netherlands and Belgium underlined the potential of spectrometric analytical methods for individual therapy planning. By investigating the volatilome of stool samples of 127 cases of neonatal late-onset sepsis and matched controls, they were able to discriminate both groups. They used FAIMS for analyzing unprocessed fecal samples of neonates which provides results after very short period of time (Berkhout et al. 2019).

Urea breath tests using radioactive carbon isotopes are the gold standard for diagnosing *Helicobacter pylori* infections and show specificity and sensitivity of approximately 95% (Nakayama and Graham 2004). With their PTR-TOF-S breathing gas investigations of infected and uninfected patients, Lechner et al. found significantly elevated hydrogen cyanide and hydrogen nitrate concentrations in *H. pylori*-infected patients (Lechner et al. 2005).

Inflammatory bowel disease diagnosis remains a clinical challenge, and Crohn’s disease and ulcerative colitis have shown distinct VOCs, which seem to reflect the gut volatilome. Breath analysis differentiated inflammatory bowel disease (IBD) from control and Crohn’s disease from ulcerative colitis (Arasaradnam et al. 2016). The same study groups were previously able to separate IBD patients from healthy controls by headspace analyses of urine samples using an e-nose and FAIMS, whereas both systems enabled discrimination of the groups (Arasaradnam et al. 2013).

### Bloodstream infections

Bacterial bloodstream infections are a common cause for sepsis, a major burden for healthcare systems with high mortality, and require fast diagnosis and therapy (SepNet Critical Care Trials Group 2016). Diagnosis still depends on unspecific clinical presentations, and pathogen identification relies on culturing, which leads to relevant delays in initiating targeted antibiotic treatment (Singer et al. 2016).

An alternative approach could be using VOC analyses of the headspace over inoculated blood cultures. Umber et al. used different GC couplings to investigate the headspace over experimental blood cultures, consisting of LB broth, human whole blood, and *E. coli*. They described six VOCs that were specific for *E. coli*-infected whole blood, even when compared to samples of inoculated LB broth without blood (Umber et al. 2013). Another group used corona discharge ionization MS to describe the increase of indole in the headspace of blood cultures incubating with *E. coli* (Zhong et al. 2019). Our own group recently published promising results regarding the use of GC-IMS for rapid differentiation of *E. coli*, *P. aeruginosa*, and *S. aureus* by analyzing the headspace of experimental blood cultures very quickly (Drees et al. 2019).

Chingin et al. conducted the first clinical feasibility study using APCI-MS (atmospheric pressure chemical ionization mass spectrometry) to investigate the volatilome of blood cultures from patients with suspected or confirmed bacteremia. Within 3–16 h, they were able to detect specific VOC patterns and successfully differentiated between five common sepsis pathogens (*Staphylococcus aureus*, *Escherichia coli*, *Klebsiella pneumoniae*, *Acinetobacter baumannii*, *and Pseudomonas aeruginosa*) (Chingin et al. 2015).

### Other applications

Urinary tract infections (UTI) are common and mostly caused by bacteria. In 2011, Storer et al. used SIFT-MS (selected ion flow tube mass spectrometry) to investigate the headspace over experimentally inoculated urine samples. They were able to distinguish between eight etiological bacteria and fungi, namely *E. coli*, *P. vulgaris*, *P. aeruginosa*, *S. aureus*, *S. epidermidis*, *K. pneumoniae*, *E. faecalis*, or *C. albicans* (Storer et al. 2011). In their clinical study from 2014, Roine et al. demonstrated the potential of an IMS based e-nose device to identify infected urine samples and to differentiate between four common causative bacteria: *E. coli*, *S. saprophyticus*, *E. faecalis*, and *Klebsiella* spp. (Roine et al. 2014).

Bacterial vaginosis is a common infection in gynecology. IMS is able to detect malodorous biogenic amines from vaginal swabs (Chaim et al. 2003). Meanwhile, IMS devices for point-of-care testing for bacterial vaginosis are commercially available and show a good accuracy (Blankenstein et al. 2015).

Infection with group B *Streptococcus* is a potentially life-threatening complication in newborn infants. By analyzing maternal vaginal swabs using a GC-IMS device, Lacey et al. recently demonstrated the ability of this method to diagnose maternal group B *Streptococcus* infections with high sensitivity and specificity. They concluded that the use of the technique as a screening tool could significantly contribute to the prevention of the condition (Lacey et al. 2020).

Recently, Daulton et al. reported of the use of a GC-IMS device to distinguish between infected and uninfected wounds. Wound infections significantly complicate the treatment of surgical patients and cause a major burden on healthcare systems worldwide. Screening for specific VOCs indicating infection of wounds could therefore be an alternative approach to the conventional microbiological diagnostic using wound swabs (Daulton et al. 2020).

By using GC–MS, Kviatkovski et al. identified 2-aminoacetophenone (2-AA) as a specific VOC indicating the presence of *P. aeruginosa* in pus samples of otitis externa patients. They assembled a device combining a whole-cell luminescent biosensor with photo-multiplier tube enabling the detection of 2-AA at very low concentrations. By using this device, they were able to diagnose otitis externa caused by *P. aeruginosa* with high accuracy (Kviatkovski et al. 2018).

## Future perspectives and challenges

During the current SARS-CoV-2 pandemic, breath tests for the detection of viral infections have come in the focus of scientific and commercial interest (Ruszkiewicz et al. 2020). This demonstrates both the potential of the methodology and the challenges that come with their application. Most of the investigations cited in this review did not identify the actual substances that contribute to a VOC pattern or fingerprint that is considered to be specific for a condition. Pattern recognition leads to valuable scientific results, and it may be justifiable to relinquish substance identification as it would be time-consuming, expensive, and in some cases impossible. The development of urgently needed diagnostic tools may be hindered in an unjustifiable manner. One may however argue that identification of a substance with a clear link to a known metabolic pathway explains its occurrence in a bacterial infectious condition and therefore increases the conclusiveness of these results. During the last decade, the value of breath tests based on spectrometric methods has been investigated in a multitude of medical conditions ranging from several lung pathologies (e.g., infections, malignomas, COPD) up to neurological conditions, such as Alzheimer’s disease (Bach et al. 2015; Darwiche et al. 2011; Fink et al. 2014). The limitation of the *pattern recognition* or *fingerprinting approach* without identification of volatile substances is the lack of causative explanation for its occurrence in the specific conditions. Most studies are monocentric, and the numbers of investigated patients are small, which increase the risk of random cohesions. In the best case, substance identification would make it possible to explain the origin of an occurring VOC by referencing the underlying mechanisms of, e.g., known bacterial metabolic pathways.

The sampling of human breath remains challenging, as breathing air by itself provides a high degree of diversity and complexity. Factors like gender, race, age, diet, and lifestyle habits can influence the composition of VOCs in human breath. Detection of a substance depends on its chemical and physical properties and on the ability to detect it with the chosen analytical method. For instance, may the ability of an IMS to detect indole over a growing *E. coli* culture depend on the type of GC column chosen for pre-separation. The reviewed literature demonstrates heterogeneous analytical approaches for the same or similar questions. It also shows that our knowledge on the biochemical implication of the occurrence or non-occurrence of VOCs in certain constellations remains limited and should be addressed in future research.

In the future, substance identification should be used to clearly link the results of basic research to the findings of clinical investigations. International and multicenter studies, adequately powered, are necessary to generate high-quality evidence for the most urgent and promising fields of application.

As it was demonstrated by Zoller and Clark a century ago, the smell of bacteria can lead to the correct diagnosis. The ability of modern spectrometric techniques makes it possible to detect smallest amounts of volatile substances and to use gas samples as a diagnostic matrix. Growing interest of the scientific community in these methods will certainly lead to more clinical applications of point-of-care diagnostics, although challenges remain.

## Conclusion

The association of volatile organic compounds (VOCs) with bacterial presence and growth has led to a novel diagnostic approach, suggesting the use of spectrometric analytical methods for the detection of pathogen-specific VOCs or VOC patterns. Basic research using complex, expensive, and space-consuming mass spectrometric methods provided knowledge on the occurrence of VOCs in association with several bacterial species. Based on these findings, less complex, robust, fast, and miniaturized applications, such as IMS and FAIMS, aim to transfer this knowledge into clinical, potentially point-of-care, use. Over the last two decades, a growing number of studies tested spectrometric analytical methods for diagnosing bacterial infections. As described in this review, there are more and more promising results demonstrating potential to supplement conventional microbiological methods, or even have the ability to substitute the latter in some selected conditions. However, future clinical research needs to be more standardized and adequately powered multicenter studies are needed to proof its value for human medicine.
